# Improving Psychological Wellbeing and Healthcare Outcomes Through Decentralization of Healthcare Expenditures in Pakistan

**DOI:** 10.3389/fpsyg.2022.882295

**Published:** 2022-10-06

**Authors:** Qurat ul Ain, Ling Xie, Tahir Yousaf

**Affiliations:** ^1^Department of Finance, School of Economics, Zhejiang University, Hangzhou, China; ^2^School of Medical Information Engineering, Zunyi Medical University, Zunyi, China; ^3^Department of Economics, School of Economics, Zhejiang University, Hangzhou, China

**Keywords:** psychological wellbeing, economics reforms, healthcare expenditures, quantitative methods, Pakistan

## Abstract

This article contributes to the limited empirical literature on the impact of decentralization on psychological wellbeing by investigating the hypothesis which signifies that shifts toward more fiscal decentralization in health services would be accompanied by improvements in health outcomes. Formulating a conventional public finance model applied to health care, this hypothesis is tested on a panel data of the Pakistan's provinces during the period 1990 to 2015. The empirical underpinning of the article suggested that the economic reforms of 2001 in Pakistan's healthcare sector, through fiscal decentralization, have imposed a substantial and positive influence on the effectiveness of the public policy in improving the healthcare outcomes over the examined period.

## Introduction

Decentralization has brought enormous changes in Fiscal structure of Pakistan since 2001 economic reforms. These reforms make a paradigm shift in social aspects of life. Though, decentralization is a wide concept but its analysis mainly analyzes the authority or structural shift. The politics and the process of devolution application require enhanced understanding to assess how federal government utilizes the decision space to bring positive change in healthcare system and future prospect challenges. Generally, people expect the future benefits and gains, the healthcare facilities, and their impact on psychological wellbeing are observed to improve (Mirowsky and Ross, 2003). The implications of decentralization have brought changes in collaboration between society and local governments social interaction, as it is mandatory to provide essential goods and services such as heath facilities. Significantly, Behera and Dash (2020) have witnessed in the Southeast Asian region from 2000 to 2014 which reveals that aggregate health expenditure, especially public health expenditure, showing positive effects on the improvement in life expectancy and reduction in infant mortality. Meanwhile, according to Behera et al. (2020), some studies elucidate the sample from Indian states between 2000 and 2016 identifying that tax revenue, economic growth, and federal transfers have a significant and positive impact on public health expenditure among the Indian states. Furthermore, a significant and positive impact of per capita tax revenue and per capita GDP on growth of public health expenditure empirically has been investigated in 16 Indian states from 1980 to 2014 (Behera and Dash, 2017).

The data of Pakistan Statistical Yearbook indicate an incremental change in total healthcare expenditures as percentage of GDP 2.79 in 2000 to 3.02 in 2015. The increasing or decreasing trend in financial support to healthcare services surely increases or reduces in individuals psychological wellbeing or psychological pressure (Banister and Hill, 2004; Zimmer and Kwong, 2004).

Certainly, devolution reforms not only contain participatory governance, local accountability, managerial efficiency, and improved responsiveness but also it can state in the form of devolution of authority to the lower-level government. The advanced forms indicate the shift of power to a parallel or lower administration level. Analysis of devolution mainly focused on structural changes, capacity of taking decisions in the provided space or achievement of better equity through performance, accountability, and efficiency. The related element as decentralization is often carried as a fundamental part of outcomes with respect to the resource redistribution, accountabilities, and responsibilities. However, the unpredictability often exists in performance, and dependent and varied perspective in a way decentralization is applied.

This study has focused on the analysis of the amelioration of psychological wellbeing through improvements in health system of Pakistan in response to the devolution process. Devolution is the most fundamental representative form of decentralization. The process of devolution was taken on by presenting a foremost constitutional amendment, and it was supported by the all major political forces to achieve the subnational demands to ensure the proper implementation and modifying the policies to the gross root level. The presented constitutional move enables provinces a more equitable portion in budget to attain solutions of the problems at local level. The purpose of this study is to comprehend to recognize and assess that how provincial governments utilize the opportunities of devolution for better planning, execution through proper spending, and further restructuring in the health systems to make it conducive for general public.

Based on this above background, the paper illustrates the following questions. Does the psychological wellbeing of general pubic of Pakistan improve or worsen due to 2001 devolution reforms? Does the outcomes of healthcare enrich by consolidating the provincial government through devolution reforms of 2001? Moreover, the prior literature is lacking in empirically examining the systematic framework of decentralization. The literature is still unable to firmly conclude that countries having more decentralized healthcare systems are producing health outcomes splendidly. The study contributes in a way by developing an empirical method to contemplate the true effect decentralized healthcare expenditures on psychological wellbeing (Section Data, Estimation Approach, and Variable Measurement). Further, this empirical approach is applied to demonstrate the above-mentioned questions (Section Empirical Results).

## Data, Estimation Approach, and Variable Measurement

### Data

To empirically test the relationship between decentralization and psychological wellbeing of citizens by providing healthcare facilities to the local citizens (Table 1 presents the details of the variables and descriptive statistics), we have used the provincial data of Pakistan and data panel consist of the period 1990–2015^1^. Fiscal data are collected from the State Bank of Pakistan, PRSP, the Pakistan Statistical Yearbooks (Pakistan Bureau of Statistics), the Pakistan economic survey (Ministry of Pakistan), and the Development statistics of Pakistan.

**Table 1:** Descriptive statistics.

**Variables**	**Definition**	**Source**	**Obs**	**Mean** ** values**	**Std.** ** values**	**Min** ** values**	**Max** ** values**
Health Exp Per capita	Measured as proportion of the Health expenditure to population of each province	*PRSP* (Ministry of Finance, 1990–2015)	104	241.29	117.48	104.31	731.91
Fully immunized children	Percentage of immunized children aged 12-23 months (taken as Log).	*Pakistan Social and Living Standard Measurement Survey* (various issues), Federal Bureau of Statistics, Government of Pakistan.	104	57.69	16.42	24	86
Number of doctors/ paramedics	Data Expressed in Thousands (Taken as Log)	Provincial Health Departments' Official Documents	104	75	15.31	2	616
Population per bed	Number of in-patient beds per 100 000 population (Taken as Log)	Handbook of Statistics on Pakistan Economy, State Bank of Pakistan	104	1,508.68	171.65	1,269	1,963
TE per capita	The ratio of provincial government expenditures to provincial population.	*Pakistan Economic survey* (Ministry of Finance, 1990-2015); *Pakistan statistical year books* (Pakistan Bureau of statistics, 1990-2015)	104	3,235.00	1,098.99	1,709.07	6,276.93
Devolution policy	N/A	N/A	104	0.538	0.501	0	1
Log GDP per capita	Log of per capita GDP	*Pakistan Economic survey* (Ministry of Finance, 1990-2015)	104	3.643	0.327	3.045	4.240
Log population	Log Population is measured as log of population value	*Pakistan statistical yearbooks* (Pakistan Bureau of statistics, 1990-2015)	104	3.254	0.872	1.709	4.657
OSR per capita	Provincial own-source revenue divided by concerned provincial population	*Pakistan Economic survey* (Ministry of Finance, 1990-2015); *Pakistan statistical year books* (Pakistan Bureau of statistics, 1990-2015)	104	3,100.95	1,026.71	1,452.85	5,888.32
Share of urban pop	It is measured as a share of urban population to the total population	*Population, Labor force and Employment* (Ministry of Finance, 1990-2015); *Pakistan statistical yearbooks* (Pakistan Bureau of statistics, 1990-2015)	104	0.316	0.133	0.158	0.585
Democratic dummy	N/A	N/A	104	0.692	0.464	0	1

### Estimation Approach

The mentioned model is used for the estimation of the impact of decentralization on PWB through its effect on health system (Equation 1), where FGLS is model used for the purpose of estimation (Lessmann, 2006; Reed and Webb, 2010; Sacchi and Salotti, 2014; Ain et al., 2020).


(1) $$\begin{array}{l} PWB_{i,t}=\alpha_{i,t}+DE_{i,t}\beta +X_{i,t}\;\gamma {+\varphi }_{i,t}+\varepsilon_{it} \end{array}$$

where *PWB*_*i, t*_ represents psychological wellbeing which here is measured by various healthcare system.

(a) Total health expenditure per capita which is represented by *HE*_*i, t*_ and shows the output indictor for the devolution efforts by the subnational government,
(b) Fully immunized children^2^ as represented by *FIC*_*i, t*_ and shows the outcome indictor for the devolution efforts by the subnational government on the health expenditures.
(c) Number of doctors/paramedics as represented by *ND*/*P*_*i, t*_ and shows the outcome indictor for the devolution efforts by the subnational government on the health expenditures.
(d) Number of population on hospital bed^3^ as represented by *NPPB*_*i, t*_ and shows the outcome indictor for the devolution efforts by the subnational government on the health expenditures.

*DE*_*i, t*_ represents the benchmark devolution reforms time period, so we take 0 before 2001 and 1 after 2001 devolution reforms,

*X*_*i, t*_ denotes the complete control variables that are taken for model estimation.

φ_*i, t*_ is used for time fixed effect and control of years.

t and i index year t and province i, respectively.

We work with a balanced panel large T dimension relative to the number of provinces (the N dimension of the panel). For this reason, the use of a dynamic panel-like system and the difference general method of moments (GMM) bias the estimators. Thus, approximating with a dynamic model does not seem to be a good empirical strategy. Moreover, according to Greene (2012), Davidson and MacKinnon (1993), and Maddala and Lahiri (2006), with many cross-sections relative to the time period, fixed and random effects can produce similar interpretations but do not allow cross-sectional correlations. However, here, we have a larger T relative to N because the panels must be balanced (and T≥m for valid results). Thus, we decided to use a cross-sectional time-series FGLS (Lessmann, 2006; Feld et al., 2010).

### Variable Measurement

#### Psychological Wellbeing

This study has taken psychological wellbeing and individual's perception as the outcome. Whereas psychological wellbeing is much broader concept. The research indicates that psychological wellbeing can be differentiated by its negative or positive effect on individual's life satisfaction (Ryff and Keyes, 1995; Hansson et al., 2008). General concept of psychological wellbeing mainly takes the following factors: happiness, life satisfaction, and psychological distress. In our study, we used a healthcare expenditures per capita and outcomes of health expenditures represented by fully immunized children, number of doctors/paramedics, and number of population on hospital bed by subnational government on the heath sector. Expenditures on heath sector improve the quality of life and improve the outcomes on health expenditures and are conceived as a higher-order factor, these facet scores create the level of psychological wellbeing, and it creates general thinking of psychological wellbeing.

#### Health Expenditures and Outcomes of Health Expenditures

Among these public services that defined the psychological wellbeing of citizens and increases their life satisfaction is healthcare facilities that is explained as healthcare expenditures by the government as the top priority (Rubio, 2011; Behera and Dash, 2020). Healthcare expenditures are measured as the percentage of total expenditures and used both development and current expenditures of hospitals and clinics, child and mother health, preventive measures and health facilities, and remaining compared to the total population of provinces. The outcome measures of healthcare expenditures are the number of paramedics and doctors, fully immunized children and number of beds in comparison with population (details are mentioned in Table 1).

#### Economic Reforms

In this study, economic reforms refer to the economic transition initiated in 2001, which has led to the fundamental changes in Pakistan. Devolution reforms of 2001 were the third reforms of decentralization process in Pakistan. These reforms were considered as more inclusive and ambitious than the previous two reforms, and it has significantly transferred the power at grassroot level. Relatively determined by the demand of highly accountable and democratic administration at local level, the Local Government Ordinance has empowered the subnational governments which were previously administered by the federal government. This process has empowered the lower-level governments as mainstream service provision provider. Therefore, in this study, we have taken 0 value for the period before 2001 and 1 for post 2001 devolution reforms following the findings of Aslam's and Yilmaz's (2011) research on the impact of decentralization reforms in Pakistan on service delivery.

#### Control Variables

In this study, GDP deflator used to deflate the per capita real GDP and 2005–2006 is taken as the base year to capture marginal diminishing welfare through incremental individual income (Jia et al., 2014). Pop is the provincial population and is taken in thousands (Bengali and Sadaqat, 2006). Share of urban population is computed by taking share of urban population in total population (Liu et al., 2017). A dummy of political is used to capture the effect of any mechanism other than decentralization that effect devolution policy. Democratic dummy takes 0 for the time period under military ruling regimes and 1 for the period that is ruled under democratic mode of Government. Following methodologies similar to those used by Baicker et al. (2012), and Jia et al. (2014), we include provincial self-financing, which is computed with the total provincial government own source revenue to provincial population by adjusting it with the GDP deflator 2005–2006 for its conversion to real prices.

## Empirical Results

**Figure 1:**
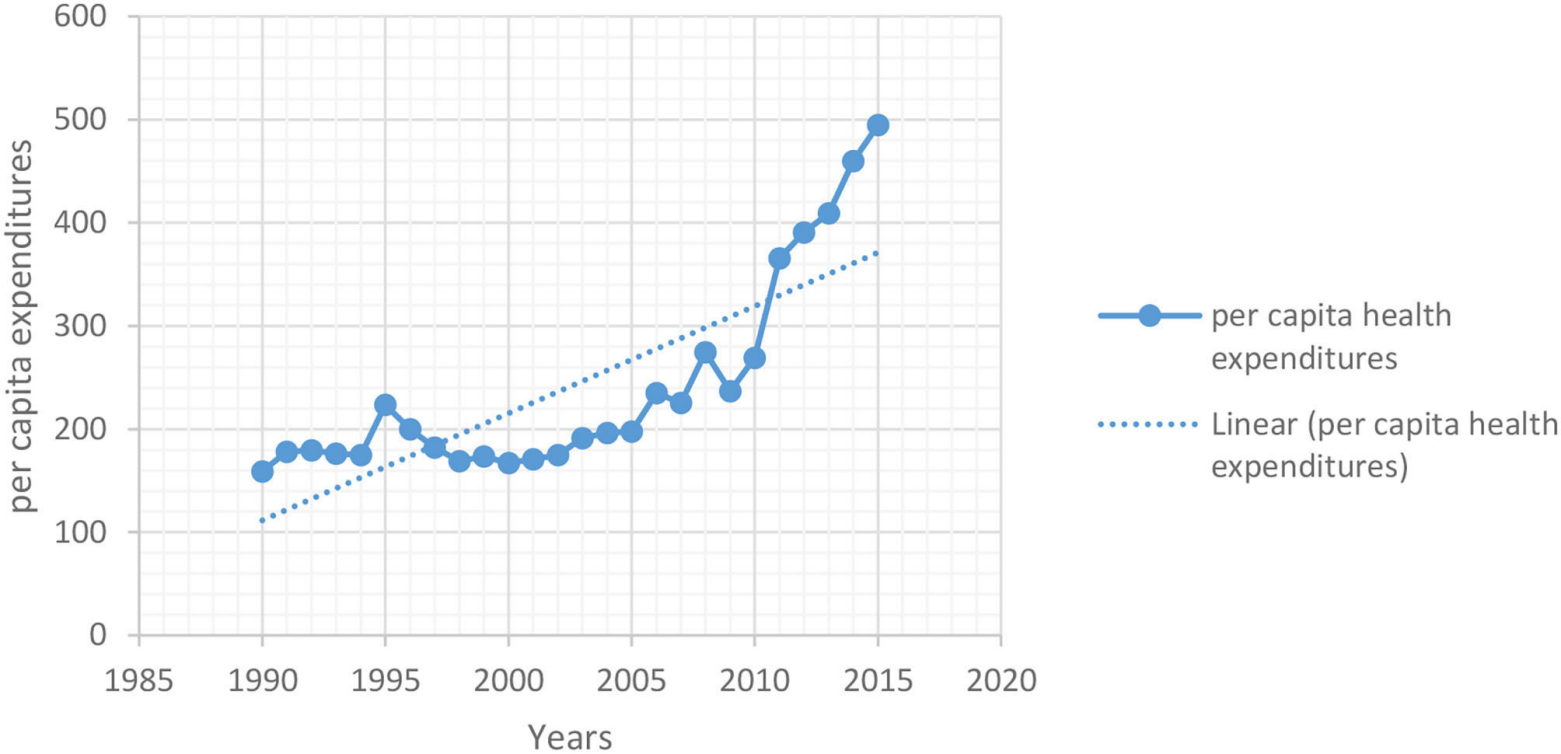
Average of provincial per capita health expenditures, 1990–2015.

Tables 2, 3 show the regression outcomes for the effects of devolution policy on psychological wellbeing of citizens by providing healthcare facilities to the local citizens. For the improvement in psychological wellbeing and social conditions for local people, health services are taken as follows: (1) health expenditures per capita (as shown in Figure 1) as output indicator and the outcome indictor include (2) immunization coverage (3) number of doctor/paramedics (4) population per bed in hospital are among the indicators used to assess the success of health policies being practiced in a country. The government expenditures on health are statistically significant and positive with devolution policy at 1% (Table 2). Alternatively, the significance of the decentralization reforms lets us determine that the magnitude of health provisions following the decentralization developments in Pakistan in 2001. Our findings are consistent with the empirical work of Jiménez-Rubio (2011), Rubio (2011), Novignon et al. (2012), and Behera and Dash (2020). A significant point is that the results remain same when we remove the endogeneity by taking the lag of regressors of independent variables as well as through 2SLS (Table 3).

**Table 2:** Effect of devolution on the per capita health expenditures and health service outcomes using FGLS.

**Dependent variable**	**Estimation method: Feasible generalized least square**			
	**Health services outcomes**			
	**Total health exp** ** (per capita exp)**	**Fully immunized children^**^a^**^**	**Number of doctors/** ** paramedics**	**Population per bed^**^b^**^**
	**(1)**	**(2)**	**(3)**	**(4)**
Devolution policy	0.0667*** (0.0263)	0.212*** (0.058)	0.121*** (0.021)	−0.197*** (0.029)
Log GDP per capita	0.102*** (0.047)	0.425*** (0.083)	0.0286** (0.008)	0.0206*** (0.005)
Log pop	0.0348** (0.0194)	0.163*** (0.037)	0.332*** (0.0421)	0.256*** (0.0326)
OSR per capita	0.385*** (0.106)	−0.0195 (0.286)	0.065 (0.977)	0.065 (0.977)
Share of urban pop	−0.236*** (0.0985)	−0.920*** (0.165)	−0.832*** (0.276)	−0.495 (0.388)
TE per capita	0.315*** (0.056)	0.278** (0.138)	0.389** (0.149)	0.386*** (0.078)
Democratic dummy	0.1227*** (0.0192)	0.122** (0.042)	0.008 (0.0184)	0.133** (0.054)
Constant	−2.061*** (0.386)	−0.146 (0.860)	1.211*** (0.0121)	1.750*** (0.012)
Wald chi square (*p* value)	341.15 (0.00)	254.57 (0.000)	267.00 (0.000)	293.00 (0.000)
Num of obs	104	104	104	104

^a^(see text footnote ^2^).

^*b*^(see text footnote ^3^).

The ^*^, ^**^, and ^***^ symbols indicate the significance at 1, 5, and 10 percent respectively.

**Table 3:** Effect of devolution on the per capita health expenditures and health service outcomes using FGLS to control endogeneity (lag of regressors and two stage least square).

**Dependent variable**	**Estimation method: Feasible generalized least square**							
	**Health services outcomes**							
	**lag of regressors**				**2SLS**			
	**(lag of regressors)** **health** **(per capita exp)**	**Fully immunized children**	**Number of doctors/** ** paramedics**	**Population per bed**	**(2sls)Health** ** (per capita exp)**	**Fully immunized children**	**Number of doctors/** ** paramedics**	**Population per bed**
	**(1)**	**(2)**	**(3)**	**(4)**	**(1)**	**(2)**	**(3)**	**(4)**
Devolution policy	0.1022*** (0.0316)	0.0951** (0.037)	0.132*** (0.031)	−0.186*** (0.028)	0.6037*** (0.1964)	0.205** (0.099)	0.904*** (0.0714)	−1.001*** (0.441)
Log GDP per capita	0.108** (0.0512)	0.499*** (0.074)	0.027** (0.009)	0.020*** (0.004)	0.1611*** (0.0623)	0.623*** (0.079)	−0.230*** (0.0889)	0.334*** (0.126)
Log pop	0.0195 (0.0222)	0.184*** (0.030)	0.223*** (0.053)	0.265*** (0.0325)	0.0988 (0.0626)	0.163*** (0.037)	0.1926*** (0.0902)	0.043*** (0.131)
OSR per capita	0.524*** (0.1399)	0.339** (0.156)	0.075 (0.978)	0.074 (0.877)	0.00014*** (0.00003)	0.613** (0.298)	1.044*** (0.256)	−0.905** (0.348)
Share of urban pop	−0.229** (0.1042)	−0.958*** (0.162)	−0.843** (0.285)	−0.585 (0.377)	−1.885*** (0.624)	−1.504*** (0.318)	−3.072*** (0.287)	2.668 (1.140)
TE per capita	0.209** (0.071)	0.1129 (0.078)	0.398** (0.159)	0.486*** (0.077)	0.00015*** (0.00005)	0.1712 (0.169)	0.180 (0.136)	−0.062 (0.139)
Democratic dummy	0.1138*** (0.0227)	0.0193 (0.0258)	0.009 (0.0195)	0.134** (0.055)	0.0606*** (0.0283)	0.0637 (0.051)	−0.1077** (0.041)	0.077** (0.037)
Constant	−1.664*** (0.487)	−0.248 (0.533)	1.321*** (0.0131)	1.540*** (0.013)	1.652*** (0.076)	−1.825** (0.792)	1.741*** (0.486)	6.568*** (1.177)
Hansen J					0.75	0.78	0.21	0.64
R-squared					0.49	0.61	0.41	0.67
Wald chi square (*p* value)	287.87 (0.00)	272.96 (0.000)	176.00 (0.000)	198.02 (0.000)	178.79 (0.00)	392.92 (0.000)	251.39 (0.00)	117.90 (0.000)
Num of obs	104	104	104	104	104	104	104	104

The ^*^, ^**^, and ^***^ symbols indicate the significance at 1, 5, and 10 percent respectively.

Immunization coverage, number of doctor/paramedics, population per bed in hospital are among the indicators used to assess the success of health policies being practiced in a country. Immunization of children is the key indicator of preventive measures and measures as the percentage of immunized children fall between the 12–23 months of age. Healthcare outcome variables from column (2)–(4) (fully immunized children, number of doctors/paramedics, and population per bed in hospital) maintain positive (negative) and strongly significant coefficient vis-à-vis the devolution reform variable, which suggests that health services have increased in after the devolution reforms. In 2001–2002, slightly more than half of children in this age group had immunization coverage that in 2014–2015. Remarkably, the findings are coherent with theoretical prospects, and the results indicate that local governments are more directed toward the needs of general public in comparison with federal government (Faguet, 2004; Watson and Khan, 2010; Yilmaz et al., 2010; Yilmaz and Venugopal, 2011).

The results of democratic dummy (1 denotes the democratic form of government and 0 otherwise) are positively associated with the health outcomes and health services. The contrary results are true for rural development. This positively emphasizes the democratic form of government. In this form, the local government elected by the local people in a democratic system is more in line with the needs of the people. Real GDP per capita is positive and significant for per capita healthcare expenditures and healthcare outcomes. Population is also an important feature that determines the scale effects in the provision of healthcare and shows positive relationship. Going forward, the allocation of government spending on public services follows a similar trend. The size of own-source income has a statistically significant positive effect on healthcare spending; however, for other some variables, the results were not statistically significant (Tables 2, 3).

### Robust Analysis

To make the results more convincing following best practices used by Kanbur and Zhang (2005), Ezcurra and Pascual (2008), Song (2013), Kyriacou et al. (2017), and Haiyue and Manzoor (2020), lagged values of a single period for all independent variables are used as regressors to avoid the issue of endogeneity (Table 3). The results again confirm the stimulatory effect of devolution policy on different components of health service expenditures. Moreover, the results support the earlier argument that subnational governments provide appropriate resources to improve their spending on the health sectors after removing endogeneity.

To mitigate this potential bias, we use a fixed-effects two-stage least squares (2SLS) approximation to assess the impact of decentralization policies on per capita health service spending and outcomes variables. This approach allows us to leverage external tools to address endogeneity. However, it requires the presence of tools that are related to endogenous variables but not directly related to the error term. According to the literature, the tools used are social security and welfare, and investment in roads and highways, which capture the mechanisms by which fiscal arrangements can influence decentralization policies.

Empirical results using the IV tool confirm the stimulating effect of decentralization policies on different components of health service spending and healthcare outcomes. Furthermore, the findings support the previous view that local governments provide appropriate resources to improve their spending in the health sector after removing endogenous factors. The Hansen J statistic for the over-identification test for instruments indicated that excluded instruments were correctly excluded, with *p*-values shown in Table 3, whereas the first-stage regressions are provided in the Table A1.

## Discussion

The Execution of devolution policy in 2001 exemplifies a major step toward decentralization of social provisions in Pakistan. The purpose of this section is to discuss decentralization from a broader view and focus on the implications of psychological wellbeing through its per capita expenditures on health services and to construct indicators to gauge the favorable or unfavorable impact of devolution in Pakistan on various outcomes of health service provision.

In Pakistan, healthcare facilities have been decentralized to local governments most commonly in past few decades, and its budgetary share is continuously growing. Therefore, improvement in this sector is perceived as a higher-order factor, the marks of these aspects produce psychological wellbeing, and it creates a broader view of psychological wellbeing (Behera and Dash, 2017, 2020; Behera et al., 2020). We have envisioned an innovative rationalization of health system authority and fiscal responsibilities to local governments. Concerning the per capita health expenditures, in both Tables 2, 3, devolution policy was specifically designed to make the health services accessible to the local citizens in a timely and efficient manner to improve the psychological wellbeing and life satisfaction.

The empirical findings of the paper propose that the 2001 devolution plan that provides higher autonomy to lower-level government was effective in providing the provisions of health service to local citizens. Similarly, the findings are similar to the work of Faguet (2004), Afaq (2007), Watson and Khan (2010), Rubio (2011), and Behera and Dash (2020) where local governments are better provider of health services because they are in better position to satisfy the general public's needs and improve the physiological wellbeing as well (Afaq, 2007).

## Conclusion

The study has examined the relationship between economic reforms which refers to the economic transition initiated in 2001 and psychological wellbeing through the healthcare facilities that is explained as healthcare expenditures using a provincial representative sample from Pakistan. The outcomes of the healthcare expenditures by the subnational government as represented by fully immunized children, number of doctors/paramedics, and number of population on hospital bed have also been examined. Our outcomes contribute a vital perception on the psychological wellbeing of the citizens by providing healthcare facilities at the regional level.

The results of the study validate that devolution is related to a substantial rise in the extent of health facilities at lower forms of government subsequent to devolution reforms promulgated in 2001. So, in general, it improves the wellbeing of local citizen. The results also demonstrate that devolution has contributed to improved health provision outcomes in the form of fully immunized children, number of doctors paramedics, and population per bed in hospitals. This is the result of lately recognized local governments giving greater importance to the distributions for local service provision from given budgets to attain advanced levels of cost efficiency in expenditures.

Recommending in terms of public policy, the results suggest that increasing healthcare expenditures, which are the part of the economic transition named devolution reform 2001 of Pakistan, are improving the local citizen's psychological wellbeing. The findings will also assist the policymakers, practitioners, and planners in scheming suitable standards and provisional strategies for fiscal allocations of transfers through devolution. Furthermore, the process of earlier consultation of transfers with provinces, i.e., delivery and expenditure capacity, projects, timeliness of transfer, and administration capacity should be commenced. The federal government institutions should focus on policymaking and its proper implementation whereas provincial governments must focus on delivery of services. Provincial governments are also required to focus on capacity building for seeking the take new tasks.

## Data availability statement

The raw data supporting the conclusions of this article will be made available by the authors, without undue reservation, to any qualified researcher.

## Author contributions

QA has done the formal analysis, methodology of the paper, and applies techniques through software. LX contributed to writing, reviewing, editing the draft, and also helped in data collection. TY has done the proof read the manuscript. All authors contributed to the article and approved the submitted version.

## Funding

We are thankful to all funding resources that facilitated us in the completion of this research. Financial support from the 2018 special project for Cultivation and Innovation of new academic, Qian Platform Talent (Grant no. [2018] 5772–012), and other resources that facilitated us in completion of this research are greatly acknowledged.

## Conflict of Interest

The authors declare that the research was conducted in the absence of any commercial or financial relationships that could be construed as a potential conflict of interest.

## Publisher's Note

All claims expressed in this article are solely those of the authors and do not necessarily represent those of their affiliated organizations, or those of the publisher, the editors and the reviewers. Any product that may be evaluated in this article, or claim that may be made by its manufacturer, is not guaranteed or endorsed by the publisher.

## Footnotes

^1^Due to data unavailability, it is limited for the period 1990–2015. The said period is taken for two possible reasons. Firstly, this period covers total 5 intergovernmental NFC awards which initiate in 1990 and most recent NFC award presented in 2015. Secondly, post 2015 data is unavailable. Four constitutional provinces are taken as federation units and data of Gilgit Baltistan and Azad Jammu Kashmir is not taken due to following two reasons. Firstly, local government governance structure is required to be operational in other parts. Secondly, data availability issue for the said period has limited us to focus on four provinces only.

^2^Percentage of children aged 12–23 months that have been immunized (based on recall or record). Note: For being classified as fully immunized, a child must have received the following vaccination: BCG, DPT1, DPT2, DPT3, Polio1, Polio2, Polio3 and Measles.

^3^Number of in-patient beds per 100,000 population.

## Appendix

**Table A1:** Estimation method FGLS: first-stage regression.

	**Health** **(per capita exp)**	**Fully immunized children**	**Number of doctors/** **paramedics**	**Population per bed**
	**(1)**	**(2)**	**(3)**	**(4)**
Log GDP per capita	0.1378 (0.1339)	0.4539*** 0.0939	0.4425*** (0.0762)	0.1562 (0.1407)
Log pop	0.1184*** (0.0436)	0.1501*** 0.0367	0.1531*** (0.0324)	0.1209*** (0.0455)
OSR per capita	1.1022*** (0.4100)	0.4654 0.4658	0.468 (0.429)	1.096** (0.4152)
Share of urban pop	−0.269 (0.256)	−0.981*** 0.208	−0.904*** (0.174)	−0.1297 (0.2559)
TE per capita	0.1530 (0.2328)	−0.1012 0.2502	−0.1945 (0.2192)	0.2633 (0.2288)
Democratic dummy	−0.417*** (0.070)	−0.4013*** 0.0652	−0.378*** (0.070)	−0.4035*** (0.0718)
Security and welfare	0.651*** (0.189)	0.0636** 0.029	0.0015*** (0.00026)	0.0624** (0.0366)
Fully immunized children	0.0624* (0.0366)			
Roads and highways		0.0017*** 0.0003	0.152*** (0.0692)	0.6655*** (0.1961)
Constant	−7.899*** (1.247)	−1.825** (0.792)	−2.634** (1.294)	−8.881*** (1.248)
R square	0.72	0.74	0.77	0.67

The ^*^, ^**^, and ^***^ symbols indicate the significance at 1, 5, and 10 percent respectively.
